# Prism adaptation theory in unilateral neglect: motor and perceptual components

**DOI:** 10.3389/fnhum.2013.00728

**Published:** 2013-11-05

**Authors:** Styrmir Saevarsson

**Affiliations:** Clinical Neuropsychology Research Group (EKN), Bogenhausen University HospitalMunich, Germany

**Keywords:** prism adaptation, motor component, perceptual component, assessment, theory, unilateral neglect

Striemer and Danckert (2010a) suggest that prism adaptation (PA) has beneficial effects primarily on spatial attention and the motor components of neglect, and that the direct effects on visual neglect are insignificant. The main support for their influential hypothesis (e.g., Saj et al., 2013) comes from their own study (Striemer and Danckert, 2010b), but Saevarsson and Kristjánsson (2013) criticize their interpretations, and call for another possible evaluation of their data. Striemer and Danckert (2013) reply to this criticism; however, there are a number of controversial and fundamental issues that remain unresolved in this debate which future empirical studies need to consider, to explain “how PA remediates symptoms of neglect” (Striemer and Danckert, 2013, p. 2).

Striemer and Danckert's (2010a) argument that PA primarily affects visuomotor and dorsal aspects of neglect, while leaving perceptual and ventral components of the syndrome mostly unaffected, underestimates the role of diagnosis and neuroanatomcial understanding of motor response deficits in unilateral neglect. Their main hypothesis is that PA improves visually guided actions but not perceptual biases that characterize neglect; however, this statement does not address the role of visual neglect in the therapeutic effects of PA. Even if the empirical findings of Striemer and Danckert (2010b) do not reveal positive effects on visual neglect, it is likely the deficit plays a passive role in improved directional motor response biases of unilateral neglect (premotor neglect: PMN). Indeed, the stronger visual unawareness is, the slower de-adaptation is, and vice versa (Michel et al., 2003, 2007; Goedert et al., 2010). Therefore, it is important to compare patients with motor response and visual neglect to visual neglect patients that do not suffer from motor aspects of neglect, in order to address the role of visual awareness following PA. Only by comparing these two groups can we explain the role of vision in PA. This contradicts Striemer and Danckert's claim that “whether the patient has been previously diagnosed as having “perceptual”or “premotor”neglect is largely irrelevant to interpreting the validity of the results.” (Striemer and Danckert, 2013, p. 2). Furthermore, Saevarsson and Kristjánsson's (2013) interpretation assumes that directional movements in neglect are improved, while visual neglect prevents de-adaptation effects. This theory does not suggest that the effects can be prevented without visual neglect, as is inexactly claimed in Striemer and Danckert (2013). They highlight a lack of available data to evaluate the validity of the theory, even though it is based largely on the same data as their similar suggestion, although Saevarsson and Kristjánsson's (2013 interpretation is different.

The subtraction method applied by Striemer and Danckert (2010b) assumes that one can subtract directional hand movements from visual perception by applying verbal landmark and manual line bisection tasks. However, it is uncertain whether this is straight forward (Saevarsson, 2013). For instance, the landmark test requires a greater cognitive load than the line bisection task: Verbal processing and more working memory items versus simple hand movements and fewer items in working memory. An increased cognitive load has been found to cause patients to “freeze” while performing (Mattingley and Driver, 1997; Husain et al., 2000). These and similar tasks requiring line bisection responses reveal inconsistent findings (e.g., Harvey et al., 2002; Harvey and Olk, 2004). Moreover, Striemer and Danckert (2010b, p 436) claim the tasks to be “perceptually equivalent.” This is questionable since the tasks are perceptually different: the landmark task is based on a pre-bisected vertical line, while line bisection is not (see e.g., Chiba et al., 2005; Saevarsson, 2013 for perceptually equivalent neglect tasks that require verbal and manual responses). Furthermore, as neglect is a multimodal deficit, it is better to test each modality one at a time, while the others are controlled, to avoid any possible misinterpretation or confounding variables (see Saevarsson, 2013 for a detailed discussion).

In support of their theory, Striemer and Danckert (2010a,b, 2013) note that neglect patients tend to gaze more often to the contralesional side following PA, although it does not produce improved perception (Dijkerman et al., 2003; Ferber et al., 2003). However, it is debated whether an increased number of contralateral eye movements in neglect following PA produces improved visual attention. Interestingly, the majority of studies maintain a contrary view (e.g., Rossetti et al., 1998; Angeli et al., 2004; Serino et al., 2006; Shiraishi et al., 2008; Vangkilde and Habekost, 2010). Additionally, Striemer and Danckert (2013) assert that they never intended to address PMN, but only motor behavior in neglect. Conversely, the response motor deficits of neglect are normally divided into two broad domains of premotor and motor neglect (e.g., Robertson and Halligan, 1999; Fink and Marshall, 2005; Saevarsson, 2013). Their study does not address motor neglect (Saevarsson, 2013), but rather directional and unilateral motor aspects of neglect (PMN). While Striemer and Danckert (2010b) measured directional motor deficit improvements with two classical PMN tasks (e.g., Bisiach et al., 1998; Harvey and Olk, 2004), the only reference they provide is Milner et al. (1993); a report on PMN (directional hypokinesia) testing which is based on a related paper from Bisiach et al. (1990). It is therefore unspecific to refer to general motor behavior aspects of the syndrome as it can, for example, refer to motor neglect, and it is uncertain how this can be applied in PA therapy and whether it differs from PMN. Further to this, Striemer and Danckert (2013) highlight that some authors doubt whether directional motor aspects of neglect are an important component of neglect syndrome (Himmelbach et al., 2007; Rossit et al., 2009). Nevertheless, most authors do not question the importance of PMN, but rather the way it is diagnosed (e.g., Mattingley and Driver, 1997; Marotta et al., 2003; Fink and Marshall, 2005; Coulthard et al., 2006; Punt and Riddoch, 2006; Goldenberg, 2010; Vallar and Mancini, 2010; Vossel et al., 2010; Loetscher et al., 2012; Saevarsson, 2013; see Saevarsson, 2013 for a detailed discussion on conceptual confusion of motor response deficits of neglect). Whether PMN belongs to the neglect syndrome or not it still needs to be addressed as it affects neglect performance.
